# Type 2 Diabetes Susceptibility in the Greek-Cypriot Population: Replication of Associations with *TCF7L2*, *FTO*, *HHEX*, *SLC30A8* and *IGF2BP2* Polymorphisms

**DOI:** 10.3390/genes8010016

**Published:** 2017-01-06

**Authors:** Christina Votsi, Costas Toufexis, Kyriaki Michailidou, Athos Antoniades, Nicos Skordis, Minas Karaolis, Constantinos S. Pattichis, Kyproula Christodoulou

**Affiliations:** 1 Department of Neurogenetics, The Cyprus Institute of Neurology and Genetics and the Cyprus School of Molecular Medicine, Ayios Dhometios, 2370 Nicosia, Cyprus; votsi@cing.ac.cy; 2 Department of Endocrinology and Diabetes, Hippocrateon Private Hospital, Engomi, 2408 Nicosia, Cyprus; c.toufexis@hippocrateon.com; 3 Department of Electron Microscopy/Molecular Pathology, The Cyprus Institute of Neurology and Genetics, Ayios Dhometios, 2370 Nicosia, Cyprus; kyriakimi@cing.ac.cy; 4 Department of Computer Science, University of Cyprus, 1678 Nicosia, Cyprus; athos.antoniades@stremble.com (A.A.); karaolis@spidernet.com.cy (M.K.); pattichi@ucy.ac.cy (C.S.P.); 5 St. George’s University Medical School at the University of Nicosia, Engomi, 2408 Nicosia, Cyprus and Department of Pediatric Endocrinology, Paedi Center for Specialized Pediatrics, Strovolos, 2025 Nicosia, Cyprus; nskordis@cytanet.com.cy

**Keywords:** type 2 diabetes, susceptibility loci, association studies, population studies, Greek-Cypriot population

## Abstract

Type 2 diabetes (T2D) has been the subject of numerous genetic studies in recent years which revealed associations of the disease with a large number of susceptibility loci. We hereby initiate the evaluation of T2D susceptibility loci in the Greek-Cypriot population by performing a replication case-control study. One thousand and eighteen individuals (528 T2D patients, 490 controls) were genotyped at 21 T2D susceptibility loci, using the allelic discrimination method. Statistically significant associations of T2D with five of the tested single nucleotide polymorphisms (SNPs) (*TCF7L2* rs7901695, *FTO* rs8050136, *HHEX* rs5015480, *SLC30A8* rs13266634 and *IGF2BP2* rs4402960) were observed in this study population. Furthermore, 14 of the tested SNPs had odds ratios (ORs) in the same direction as the previously published studies, suggesting that these variants can potentially be used in the Greek-Cypriot population for predictive testing of T2D. In conclusion, our findings expand the genetic assessment of T2D susceptibility loci and reconfirm five of the worldwide established loci in a distinct, relatively small, newly investigated population.

## 1. Introduction

Type 2 diabetes (T2D) is a chronic complex heterogeneous disease of glucose metabolism caused by multiple genetic, epigenetic and environmental factors [1]. It is characterised by high blood glucose levels caused by the combination of insulin resistance and impaired insulin secretion [2,3]. It is a serious worldwide public health burden which has reached epidemic proportions with an increasing prevalence and substantial familial clustering [4]. The disease leads to morbidity with the life expectancy being reduced, while additional implications include premature coronary heart disease, peripheral vascular disease, renal failure, stroke and amputation [2].

The heritability of the disease has been well established through twin and family studies [5] that also revealed an estimated lifetime risk of 38% by the age of 80 if one parent is affected and by the age of 60 if both parents are affected [6]. T2D has been the subject of numerous genetic studies aiming at the elucidation of the genetic mechanisms involved in the development of the disease. Linkage and candidate gene association studies initially reported many T2D linked chromosomal regions. However, only *PPARG*, *KCNJ11* and *TCF7L2* were replicated in most populations, thus being established as T2D associated genes [2,4]. Development of the high-throughput single nucleotide polymorphism (SNP) genotyping technology and completion of the HapMap project enabled the implementation of genome-wide association studies (GWAS) [7] and since 2007 they have become the leading tool for the identification of several T2D susceptibility loci [2,8]. Recent GWAS, initially performed in Caucasians and then in non-European populations, and meta-analyses of these studies, have increased the total number of the identified variants with possible association to over 88 variants, including and further confirming some variants that were previously identified by the linkage and candidate gene studies [9,10]. However, additional variants are likely to be discovered since the identified associations explain only about 10% of the heritability of the disease [2,4,8].

Further to GWAS, replication studies have been performed in various populations which aimed at investigating whether identified T2D susceptibility variants confer risk across different genetic backgrounds [4,11,12,13,14,15,16]. In this concept, we performed the first genetic study on T2D in the Greek-Cypriot population, which represents the great majority (74%) of the Cypriot population (694,700 indigenous individuals at the 2014 census) [17] and has proved to be genetically distinct through other genetic studies, such as on Thalassaemia [18] and breast cancer susceptibility [19]. The prevalence of diabetes in the adult Greek-Cypriot population was estimated to be 10.3% [20] in 2006, which is close to the global prevalence and more similar to that of Middle East and North African populations rather than European populations (International Diabetes Atlas, [21]). This pilot replication study initiates evaluation of T2D susceptibility loci in the Greek-Cypriot population. Results for 21 SNPs that were associated with T2D in other populations until the year 2010 are presented. 

## 2. Materials and Methods

### 2.1. Study Participants

A total of 1018 subjects, including 528 unrelated well characterised Greek-Cypriot T2D patients and 490 controls without diabetes, were recruited for this study. The established World Health Organization (WHO) diagnostic criteria were used for the diagnosis of T2D. Control participants’ selection criteria included fasting plasma glucose levels of ≤108 mg/dL and absence of a T2D family history. T2D is a relatively late onset disease. In order to minimize the possibility of including individuals that will later develop T2D, we mostly selected individuals above the age of 50 for the study control group. 

Glucose levels were recorded for all study participants. Clinical, biochemical data (Table 1) and other information such as age of T2D first diagnosis, specific diet, medication or complications of the disease, including cardiovascular disease (CVD), ophthalmological symptoms, hypertension and nephropathy were also recorded for the majority of the study participants. The study was conducted in accordance with the Declaration of Helsinki, and the ethical approval was granted by the National Bioethics Committee of Cyprus (ΕΕΒΚ/ΕΠ/2010/19, 8 March 2011). Written informed consent was obtained from all study participants.

### 2.2. DNA Extraction

DNA was extracted from whole blood using standard salting out procedures. DNA samples were diluted and aliquots were plated in duplicates in 384-deep-well storage plates at a uniform concentration of 10 ng/μL.

### 2.3. SNP Selection and Genotyping

Twenty-one SNPs associated with T2D in other populations until year 2010 have been investigated in this study (Table 2). SNPs were selected through a literature search of T2D GWAS and from candidate gene studies [22,23,24,25,26,27,28,29,30]. Samples were genotyped using pre-designed TaqMan SNP Genotyping Assays following the standard protocol provided by the manufacturer (Applied Biosystems, Foster City, CA, USA). The genotyping success rate was >99%.

### 2.4. Statistical Analyses 

Student’s *t*-test was used to evaluate the differences of the continuous variables (presented as mean ± standard deviation) between cases and controls. Quality control (QC) checks were performed, for samples and SNPs. We evaluated the genotype distributions of all SNPs in the control samples for Hardy–Weinberg equilibrium (HWE) using an exact test [31] with a threshold of deviation *p* ≤ 0.05. Minor allele frequencies (MAF) were calculated and used for quality control (variants with MAF < 0.01 were excluded). Linear (for T2D age of onset) and logistic regression (for T2D) analyses were performed for samples and SNPs passing the QC filters. Analyses were performed in R (R Development Core Team, Vienna, Austria) [32] and PLINK (Center for Human Genetic Research, Boston, MA, USA) [33,34]. Logistic regression analyses were performed at two levels: (a) with an adjustment for age and gender; and (b) with an adjustment for age, gender and Body Mass Index (BMI). Statistical significance for T2D association with the tested variants was defined using a *p*-value threshold of 0.05. Linear regression analysis was performed with an adjustment for gender and BMI. Bonferroni’s method was applied for multiple testing correction to determine the significance of potential novel associations with T2D age of diagnosis, using a *p*-value threshold of 0.0026 (0.05/19).

## 3. Results

### 3.1. Study Participants

The main phenotypic characteristics of the study participants are presented in Table 1. The mean age of patients and controls at the time of interviewing and sampling was slightly different (63.73 and 59.14 respectively). However, the great majority was over 50 which is close to the mean age of T2D diagnosis (52.08). Overall, the mean values of the remaining characteristics—BMI, glucose, High-density lipoprotein cholesterol (HDL-C), Low-density lipoprotein cholesterol (LDL-C), Total Cholesterol (TC), and Triglycerides (TG)—were all significantly different between the two groups with the most significant difference observed at the glucose levels (*p* = 1.1 × 10^−120^). Patients had significantly higher BMI, glucose and TG and lower HDL, LDL and TC levels compared to the controls. The observed differences in the lipid patterns between the two groups may be attributed to targeted LDL lowering medication taken by the patients.

### 3.2. Association Studies—Statistical Analyses

Twenty-one SNPs were analysed (Table 2). All SNPs, except rs12779790 and rs757210, passed QC. SNP rs12779790 failed HWE in controls (*p* < 0.05). SNP rs757210 was found to be tri-allelic in our study, a finding that was confirmed by other studies [35,36], and was thus not used in the analyses. Linkage disequilibrium (LD) testing confirmed that none of the study SNPs was in LD.

The 19 SNPs were tested for association with T2D and the results are presented in Table 2 and Supplementary Table S1. Effects and confidence intervals reported in previous studies are also presented in Table 2. The current study results were aligned in order to correspond to the published risk allele. Initial analysis was performed with an adjustment for age and gender, which resulted in four loci showing statistically significant associations with T2D (Supplementary Table S1) at nominal significance levels (*p* < 0.05); *TCF7L2* rs7901695 [odds ratio (OR) (95% confidence interval (CI)) 1.3 (1.08–1.55) *p*-value = 0.005], *FTO* rs8050136 [OR (95% CI) 1.34 (1.11–1.61) *p*-value = 0.002], *HHEX* rs5015480 [OR (95% CI) 1.36 (1.13–1.62) *p*-value = 0.001] and *SLC30A8* rs13266634 [OR (95% CI) 1.31 (1.08–1.6) *p*-value = 0.007]. Further analysis with adjustment for age, gender and BMI showed statistically significant associations with five variants; in addition to the previous four, association with locus *IGF2BP2* rs4402960 [OR (95% CI) 1.24 (1.01–1.53) *p*-value = 0.04] was also observed (Table 2). Furthermore, adjustment for BMI slightly strengthened the T2D association with *TCF7L2* [OR (95%CI) 1.35 (1.1–1.64) *p*-value = 0.003], slightly weakened the association with *HHEX* [OR (95%CI) 1.38 (1.13–1.69) *p*-value = 0.002] and *FTO* [OR (95% CI) 1.33 (1.08–1.63) *p*-value = 0.006] and significantly weakened the association with *SLC30A8* [OR (95% CI) 1.31 (1.05–1.63) *p*-value = 0.02]. 

Association analyses were also performed between the 19 SNPs and age of T2D diagnosis using linear regression and adjusting for gender and BMI (Table 3). Although two variants (*THADA* rs 7578597 and *TCFL2* rs7901695) were associated with earlier age of diagnosis at nominal significance levels (*p* < 0.05), these did not survive Bonferroni correction [*p* < 0.0026 (0.05/19)]. For each extra risk allele of SNP rs7578597, the mean age of diagnosis was reduced by 3.81 years (*p* = 0.006) and for each extra risk allele of rs7901695, the mean age of diagnosis was reduced by 1.91 years (*p* = 0.005).

## 4. Discussion

Type 2 Diabetes is a chronic complex disease of glucose metabolism with a strong genetic contribution and a worldwide prevalence that reaches epidemic proportions [2,4]. Many GWAS [24,27,37], meta-analyses [2,5,23,26] and replication studies have been performed that focused on common variants in different ethnic groups [4,12,38] and identified a large number of SNPs that are associated with the disease. Substantial genetic heterogeneity has been observed between the different populations suggesting that exploration of T2D susceptibility in additional populations might provide further insight into the disease aetiology.

We performed the first genetic study of T2D in the Greek-Cypriot population, a population of Caucasian origin. Compared to other Caucasian populations the Cypriot population is genetically distinct with significant differences from the Northern European populations [39,40], and likely has genetic similarities with the current populations of Levant [41]. Previous studies revealed unique genetic features in this population [18,19] and in one of them, the genetic characteristics of Greek- and Turkish-Cypriots were compared with the mainland Greek and Turkish populations. This study revealed a close genetic similarity between the two Cypriot communities and considerable differences with the Greek and Turkish populations [18]. The island, inhabited by the Greeks during the Bronze age, is located at the crossroads of three continents (Africa, Asia, and Europe) and has come under the domination of successive foreign invaders in its long history; including the Phoenicians, the Assyrians, the Egyptians, the Persians, the Romans, the Arabs, the Franks, the Venetians, the Ottoman Turks and the British. This pool of interactions with other populations has probably led to the assimilation of not only cultural influences but also genetic influences, thus contributing to the genetic background of the Cypriot population.

This study focused on an initial evaluation of 21 known T2D common susceptibility loci, most of which were identified in Caucasian populations. Five of the tested loci (rs7901695 in *TCF7L2*, rs8050136 in *FTO*, rs5015480 near *HHEX*, rs13266634 in *SLC30A8* and rs4402960 in *IGF2BP2*) showed statistically significant associations with T2D in this population after adjustment for age, gender and BMI. Previous studies on SNP rs8050136 (*FTO*) report that the association with T2D is abolished after adjustment for BMI, thus indicating that the association is probably mediated through a primary effect on BMI, a finding that necessitates the inclusion of BMI as a covariate in similar analyses [22,23,38,42,43]. In this study, adjustment for BMI weakened, but did not abolish the association of rs8050136 (*FTO*) with T2D. This result indicates that the association is likely not mediated through BMI in the Greek-Cypriot population, also in agreement with other published studies [44,45].

Nine of the non-statistically significant variants are affecting the disease in the same direction as previously reported in other populations, suggesting that these associations might not have been identified in this population due to reduced power. However, lack of association may also be attributed to true genetic diversity. At least for the significant loci and for the majority of the loci affecting the disease in the same direction, the obtained risk allele frequencies (RAF) and effect sizes in the Greek-Cypriot population, are similar, with overlapping confidence intervals with the published studies. The highest difference in the obtained RAF compared to the published study data was observed for SNP rs2237892 (*KCNQ1*), which was initially identified in the Japanese population [29] (Japanese RAF = 0.61, Greek-Cypriot RAF = 0.96). However, the equivalent reported European RAF is 0.93 [29], which is close to the RAF obtained in the present study. The trend of 14/19 SNPs to affect the disease in the same direction suggests that these variants can have a potential predictive value in the Greek-Cypriot population. Future investigation targeting a larger sample size and a larger selection of SNPs is expected to overcome the current limitations and enable the genetic characterization of T2D susceptibility in this population. Furthermore, a genome-wide study might reveal novel variants, thus expanding the knowledge on genetic susceptibility of T2D. 

The association of age at T2D diagnosis with the 19 SNPs was further tested. Previous studies in other populations revealed associations of the age at T2D first diagnosis with specific loci, including variants in the *TCF7L2*, *FTO* and *TMEM* genes [22,44,46,47]. In this study, significant associations of age at T2D diagnosis with SNPs rs7578597 (*THADA*) and rs7901695 (*TCF7L2*) were detected at nominal significance, however both associations did not survive Bonferroni correction.

To our knowledge, similar replication studies in neighbouring populations have only been performed in the Lebanese population [12,48,49] and more recently, a T2D GWAS has also been reported [50]. In the Lebanese population two of the replicated associations (rs7901695 in *TCF7L2* and rs4402960 in *IGF2BP2*) are common with replicated associations in the Greek-Cypriot population. Additional SNPs (rs13266634 in *SLC30A8* and rs8050136 in *FTO*) replicated with significant association in this study, were not statistically associated with T2D in the Lebanese population [12]. The Lebanese replication studies did not include an analysis of the fifth replicated association (rs5015480 in *HHEX*) in the Cypriot population. Furthermore, a lack of association in the Lebanese population is reported for five of this study’s non-significant SNPs (rs864745, rs7578597, rs10923931, rs4607103, rs10010131) [12], for three of which (rs864745, rs7578597 and rs4607103) the currently obtained effect sizes are in the opposite direction compared to the European GWAS. In the recent Lebanese GWAS study, leading variants in two loci (rs7766070 in *CDKAL1* and rs34872471 in *TCF7L2*) were reported with genome-wide significant association [50].

A few small studies relevant to T2D susceptibility or to disease complications have been reported in the Greek population focused on loci that were not identified through GWAS [51,52]. Association of T2D with *C1q* SNP rs2920001 [51] and diabetic nephropathy with *IL-6* SNP rs1800795 were identified in the Greek population [52]. In a third study, five of the established T2D susceptibility loci were tested to examine if Gestational Diabetes Mellitus (GDM) exhibits a genetic predisposition similar to that of T2D [53]. An association of GDM with two of these loci, including a *TCF7L2* gene variant (rs7903146) was obtained through the study. Overall, published studies do not allow for a comparison between the Greek and the Greek-Cypriot populations due to the absence of any common study SNPs or diabetes study type.

A replication study has not been performed in any of the remaining neighbouring populations. The majority of studies in the Turkish population were focused on variants in single genes, which identified the association of T2D with *CAPN10* variants [54,55], an *IRS1* variant [56], some *ABCC8* variants [57] and some *ADIPOQ* variants [58]. In one study, a lack of association was reported for the *KCNJ11* variant rs5219 [57], in agreement with the lack of association also reported in the Greek-Cypriot population. In the Egyptian population, T2D susceptibility was associated with *MTHFR* [59], *IL-4*, *IL-13* [60] and *GSTP1* [61] variants. Overall, published studies in both the Turkish and the Egyptian populations do not allow for a comparison with the Greek-Cypriot population because different SNPs were investigated in each study. 

The majority of T2D susceptibility loci including four of the current study replicated loci (*TCF7L2*, *HHEX*, *SLC30A8* and *IGF2BP2*), have been associated with impairments in insulin secretion or sensitivity [4,62]. In addition, some loci, including two of the current study replicated loci (*FTO* and I*GF2BP2*), have been associated with regulation of adipogenesis [45,63]. However, less information exists on the molecular mechanisms through which the associated SNPs can alter the function of each gene. Previous studies on candidate genes and the DNA methylation profiling in pancreatic islets from patients and controls, demonstrated a key role for epigenetic modifications in T2D pathogenesis [64,65,66,67]. Epigenetic modifications have more recently been proposed as a potential mechanism through which the associated SNPs can alter the normal function of a T2D candidate gene as well. Therefore, an interaction of genetic and epigenetic mechanisms affecting T2D susceptibility may be implicated in the molecular mechanisms through which susceptibility SNPs alter the function of each gene [1,68]. A recent study was focused on 19 of the T2D associated SNPs that introduce or delete possible Cytosine-phosphate-Guanine (CpG) methylation sites, including three of the statistically significant SNPs associated with T2D in our study (rs7901695, rs5015480 and rs13266634). All tested CpG SNPs have been associated with differential methylation of these sites in human non-diabetic pancreatic islets and some of them, including rs7901695, with differential methylation of surrounding CpG sites as well. In addition, some of these SNPs exhibiting differential DNA methylation, were associated with altered gene expression, alternative splicing events (including rs13266634 and rs7901695) and hormone secretion (including rs5015480) in the human islets [1]. A more recent study on genome-wide DNA methylation of pancreatic islets showed that DNA methylation patterns of T2D candidate genes, including the *TCF7L2*, *FTO* and *HHEX*, were altered in human islets from patients with T2D compared to controls without diabetes. New target genes with altered DNA methylation and expression in human T2D islets contributing to impaired insulin and glucagon secretion, have also been identified [68].

## 5. Conclusions

This study initiates the genetic assessment of T2D in a distinct newly investigated population. Statistically significant associations of T2D with five established loci (*TCF7L2*, *FTO*, *HHEX*, *SLC30A8* and *IGF2BP2*) are replicated in the Greek-Cypriot population. Fourteen of the nineteen loci tested had ORs in the same direction as previously published, suggesting that these variants can potentially be used in the Greek-Cypriot population for predictive testing of T2D. 

## Figures and Tables

**Table 1:** Summary of the main phenotypic characteristics of the study participants.

Trait	T2D Patients	Controls	*t*-Test *p*-Value
**Number**	528	490	n/a
**Sex (male/female)**	321/207	263/227	n/a
**Age at interview (years, mean ± SD)**	63.73 ± 10.50	59.14 ± 11.91	1.1 × 10^−10^
**Age at diagnosis (years, mean ± SD)**	52.08 ± 11.01	n/a	n/a
**BMI (kg/m^2^ ± SD)**	30.02 ± 4.95	26.75 ± 4.03	1.3 × 10^−25^
**Glucose (mg/dL ± SD)**	149.63 ± 48.37	89.11 ± 9.11	1.1 × 10^−120^
**HbA1c (%) (DCCT ± SD)**	0.17 ± 0.90	n/a	n/a
**HDL-C (mg/dL ± SD)**	43.35 ± 12.23	51.52 ± 14.91	2.0 × 10^−19^
**LDL-C (mg/dL ± SD)**	102.39 ± 31.18	136 ± 34.29	7.3 × 10^−50^
**TC (mg/dL ± SD)**	176.11 ± 40.42	208.68 ± 40.47	2.9 × 10^−33^
**TG (mg/dL ± SD)**	149.66 ± 86.11	118.73 ± 64.46	5.8 × 10^−10^

T2D: Type 2 diabetes; BMI: body mass index; HbA1c: Haemoglobin A1c; HDL-C: high-density lipoprotein cholesterol; LDL-C: low-density lipoprotein cholesterol; TC: total cholesterol; TG: triglycerides.

**Table 2:** Summary of the single nucleotide polymorphisms (SNPs) included in the study.

SNP	Nearest Gene(s)	Chromosome	Reference	Non Risk/Risk Allele ^a^	RAF ^b^	OR ^b^ (95% CI)	Frequency ^c^	OR ^c^ (95% CI)	*p*-Value ^d^
				Published Results			Current Analysis		
**rs10923931**	*NOTCH2*	1p12	[23]	G/T	0.11	1.13 (1.08–1.17)	0.06	1.09 (0.73–1.64)	0.68
**rs7578597**	*THADA*	2p21	[23]	C/T	0.90	1.15 (1.10–1.20)	0.94	0.75 (0.49–1.15)	0.19
**rs4607103**	*ADAMTS9*	3p14.1	[23]	T/C	0.76	1.09 (1.06–1.12)	0.59	0.89 (0.73–1.1)	0.29
**rs4402960**	*IGF2BP2*	3q27.2	[24]	G/T	0.30	1.14 (1.11–1.18)	0.27	1.24 (1.01–1.53)	**0.04**
**rs1801282**	*PPARG*	3p25.2	[24]	G/C	0.82	1.14 (1.08–1.20)	0.95	1.33 (0.82–2.16)	0.25
**rs10010131^e^**	*WFS1*	4p16.1	[25]	A/G	0.60	1.16 (1.05–1.28)	0.66	1.2 (0.97–1.49)	0.09
**rs4457053**	*ZBED3*	5q13.3	[26]	A/G	0.26	1.08 (1.06–1.11)	0.31	0.96 (0.77–1.19)	0.69
**rs10946398**	*CDKAL1*	6p22	[22]	A/C	0.32	1.16 (1.10–1.22)	0.32	1.21 (0.99–1.49)	0.07
**rs864745**	*JAZF1*	7p15.1	[23]	G/A	0.50	1.10 (1.07–1.13)	0.55	0.82 (0.67–1)	0.05
**rs13266634**	*SLC30A8*	8q24.11	[24]	T/C	0.61	1.12 (1.07–1.16)	0.69	1.31 (1.05–1.63)	**0.02**
**rs10811661**	*CDKN2A*	9p21	[27]	C/T	0.83	1.20 (1.12–1.28)	0.79	1.13 (0.87–1.46)	0.35
**rs12779790^f^**	*CDC123*, *CAMK1D*	10p13	[23]	A/G	0.18	1.11 (1.07–1.14)	-	-	-
**rs5015480**	*HHEX*	10q23.33	[22]	T/C	0.57	1.13 (1.07–1.19)	0.53	1.38 (1.13–1.69)	**0.002**
**rs7901695**	*TCF7L2*	10q25.2	[22]	T/C	0.27	1.37 (1.25–1.49)	0.41	1.35 (1.1–1.64)	**0.003**
**rs10830963**	*MTNR1B*	11q14.3	[28]	C/G	0.27	1.09 (1.05–1.12)	0.25	1.13 (0.9–1.41)	0.29
**rs5219**	*KCNJ11*	11p15.1	[24]	C/T	0.46	1.14 (1.10–1.19)	0.33	0.95 (0.77–1.19)	0.67
**rs2237892**	*KCNQ1*	11p15.5	[29]	T/C	0.61	1.45 (1.34–1.47)	0.96	1.25 (0.7–2.23)	0.44
**rs7961581**	*TSPAN8*, *LGR5*	12q21.1	[23]	T/C	0.27	1.09 (1.06–1.12)	0.41	1.02 (0.83–1.26)	0.84
**rs8042680**	*PRC1*	15q26.1	[26]	C/A	0.33	1.07 (1.05–1.09)	0.45	1.09 (0.88–1.33)	0.43
**rs8050136**	*FTO*	16q12.2	[22]	C/A	0.41	1.23 (1.18–1.32)	0.41	1.33 (1.08–1.63)	**0.006**
**rs757210^f^**	*HNF1B*	17q12	[30]	G + C/A	0.38	1.12 (1.07–1.18)	-	-	-

^a^ Non risk/ Risk allele based on the published study; ^b^ Published risk allele frequency (RAF) odds ratio (OR) and 95% confidence interval (CI); ^c^ RAF, OR and 95% CI obtained in the current study population; ^d^ *p*-value from logistic regression adjusted for age, gender and BMI. A *p*-value threshold of 0.05 was used and the identified significant associations are shown in bold; ^e^ OR converted to be in respect of the risk allele; ^f^ Failed quality control in this study.

**Table 3:** Results for single SNP association analyses with the age at T2D first diagnosis.

SNP	Gene	Beta	Standard Error	*p*-Value
rs10923931	*NOTCH2*	0.48	1.38	0.729
rs7578597	*THADA*	−3.81	1.38	**0.006**
rs4607103	*ADAMTS9*	−0.98	0.71	0.172
rs4402960	*IGF2BP2*	−0.64	0.73	0.376
rs1801282	*PPARG*	−1.97	1.81	0.277
rs10010131	*WFS1*	−0.54	0.75	0.473
rs4457053	*ZBED3*	−0.41	0.77	0.595
rs10946398	*CDKAL1*	−0.31	0.72	0.671
rs864745	*JAZF1*	−0.34	0.70	0.632
rs13266634	*SLC30A8*	0.25	0.80	0.753
rs10811661	*CDKN2A*	0.47	0.95	0.620
rs5015480	*HHEX*	0.26	0.71	0.713
rs7901695	*TCF7L2*	−1.91	0.67	**0.005**
rs10830963	*MTNR1B*	−0.22	0.77	0.774
rs5219	*KCNJ11*	−0.25	0.77	0.743
rs2237892	*KCNQ1*	−0.55	2.08	0.792
rs7961581	*TSPAN8*, *LGR5*	0.33	0.73	0.655
rs8042680	*PRC1*	−0.66	0.74	0.376
rs8050136	*FTO*	−0.60	0.70	0.393

Regression estimates and *p*-values based on linear regression adjusted for gender and BMI. Nominally significant associations (*p* < 0.05) are shown in bold, however they did not survive Bonferroni correction (*p* < 0.0026).

## Supplementary Materials

The following is available online www.mdpi.com/2073-4425/8/1/16/s1, Table S1: T2D association logistic regression analysis results obtained with adjustment for age and gender or age, gender and BMI.
