# Supplementary material for: Type 2 Diabetes Susceptibility in the Greek-Cypriot Population: Replication of Associations with TCF7L2, FTO, HHEX, SLC30A8 and IGF2BP2 Polymorphisms
Source: Genes (Basel). 2017 Jan 6;8(1):16. doi: 10.3390/genes8010016 (PMC5295011; doi:10.3390/genes8010016)
Supplement: Supplementary file 1 [file genes-08-00016-s001.pdf]

Supplementary Materials: Type 2 Diabetes Susceptibility in the Greek-Cypriot Population: Replication of Associations with *TCF7L2, FTO, HHEX, SLC30A8* and *IGF2BP2* Polymorphisms

Christina Votsi, Costas Toufexis, Kyriaki Michailidou, Athos Antoniades, Nicos Skordis, Minas Karaolis, Constantinos S. Pattichis and Kyproula Christodoulou

Table S1. Type 2 Diabetes (T2D) association logistic regression analysis results obtained with adjustment for age and gender or age, gender and body mass index (BMI).

SNP	Nearest Gene(s)	OR (95% CI)	*p*-Value	OR (95% CI)	*p*-Value
		Adjusted for Age and BMI		Adjusted for Age, Gender and Gender	
rs10923931	*NOTCH2*	1.22 (0.84–1.78)	0.297	1.09 (0.73–1.64)	0.68
rs7578597	*THADA*	0.76 (0.52–1.12)	0.161	0.75 (0.49–1.15)	0.19
rs4607103	*ADAMTS9*	0.92 (0.77–1.11)	0.386	0.89 (0.73–1.1)	0.29
rs4402960	*IGF2BP2*	1.17 (0.97–1.42)	0.103	1.24 (1.01–1.53)	**0.04**
rs1801282	*PPARG*	1.15 (0.75–1.76)	0.530	1.33 (0.82–2.16)	0.25
rs10010131 [a]	*WFS1*	1.14 (0.94–1.38)	0.175	1.2 (0.97–1.49)	0.09
rs4457053	*ZBED3*	0.96 (0.79–1.17)	0.709	0.96 (0.77–1.19)	0.69
rs10946398	*CDKAL1*	1.23 (1.02–1.49)	0.033	1.21 (0.99–1.49)	0.07
rs864745	*JAZF1*	0.87 (0.72–1.04)	0.121	0.82 (0.67–1)	0.05
rs13266634	*SLC30A8*	1.31 (1.08–1.6)	**0.007**	1.31 (1.05–1.63)	**0.02**
rs10811661	*CDKN2A*	1.21 (0.95–1.53)	0.120	1.13 (0.87–1.46)	0.35
rs12779790 [b]	*CDC123,*	-	-	-	-
rs5015480	*HHEX*	1.36 (1.13–1.62)	**0.001**	1.38 (1.13–1.69)	**0.002**
rs7901695	*TCF7L2*	1.3 (1.08–1.55)	**0.005**	1.35 (1.1–1.64)	**0.003**
rs10830963	*MTNR1B*	1.07 (0.88–1.31)	0.485	1.13 (0.9–1.41)	0.29
rs5219	*KCNJ11*	0.9 (0.74–1.09)	0.272	0.95 (0.77–1.19)	0.67
rs2237892	*KCNQ1*	1.22 (0.72–2.08)	0.463	1.25 (0.7–2.23)	0.44
rs7961581	*TSPAN8, LGR5*	1.01 (0.84–1.22)	0.918	1.02 (0.83–1.26)	0.84
rs8042680	*PRC1*	1.09 (0.91–1.32)	0.346	1.09 (0.88–1.33)	0.43
rs8050136	*FTO*	1.34 (1.11–1.61)	**0.002**	1.33 (1.08–1.63)	**0.006**
rs757210b	*HNF1B*	-	-	-	-

[a] Odd ratio (OR) converted to be in respect of the risk allele; [b] Failed quality control in this study; A *p*-value threshold of <0.05 was used; identified significant associations are shown in bold. SNP: single nucleotide polymorphism; CI: Confidence interval.
